# Research on Data Security Communication Scheme of Heterogeneous Swarm Robotics System in Emergency Scenarios

**DOI:** 10.3390/s22166082

**Published:** 2022-08-14

**Authors:** Yi Sun, Ying Shao

**Affiliations:** 1 College of Communication and Information Engineering, Xi’an University of Science and Technology, Xi’an 710054, China; 2 Xi’an Key Laboratory of Heterogeneous Network Convergence Communication Affiliation, Xi’an 710054, China

**Keywords:** heterogeneous swarm robotics, blockchain, DPoS consensus mechanism, Byzantine generals problem, data security

## Abstract

In emergency scenarios where the on-site information is completely lacking or the original environmental state has been completely changed, autonomous and mobile swarm robotics are used to quickly build a rescue support system to ensure the safety of follow-up rescuers and improve rescue efficiency. To address the data security problem caused by the complex and changeable topology of the heterogeneous swarm robotics network in the process of building the rescue support system, this paper introduced a decentralized data security communication scheme for heterogeneous swarm robotics. First, we built a decentralized network topology model by using base robot, communication robotics, and business robotics, and it can ensure the stability of the system. Moreover, based on the decentralized network topology model, we designed a storage model using the master–slave blockchain method. The master chain is composed of base robot and communication robotics, which mainly store the digests of robot data in multiple slave chains to reach the global data consensus of the system. The slave chains are composed of business robotics and communication robotics, which mainly store all data on the slave chains to reach the local data consensus of the system. The whole data storage system adopts the Delegated Proof of Stake consensus mechanism to elect proxy nodes to participate in the data consensus tasks in the system and to ensure the data consistency of each robot node in the decentralized network. Additionally, a prototype of the heterogeneous swarm robotics system based on the master–slave chains is constructed to verify the effectiveness of the proposed model. The experimental results show that the scheme effectively solves the data security problem caused by the unstable communication link of the heterogeneous swarm robotics system.

## 1. Introduction

Robots are becoming more and more important in human society because of their mobility, wide application and ability to perform high-risk tasks. In an unknown post-disaster rescue scenario, the robot can perform an initial search to locate survivors and collect information about their survivors’ physical conditions [1] so that subsequent rescuers can perform rescue tasks quickly and efficiently. However, due to the destruction of the basic communication facilities in the unknown environment, the robot system must be autonomous and mobile. For example, Cai L. [2] used multiple mobile robots to complete the search in the unknown indoor scene through cooperation in a rescue mission. The swarm robotics system has the characteristics of the typical distributed system and the multi-agent system, and the system consists of multiple robots with simple structures and specific functions. Each robot interacts with the others, quickly adapts to the dynamic environment, and jointly completes specific tasks [3,4]. Different from the sensor network [5], the robot in the swarm robotics system can control its own behavior and complete specific tasks according to its local perception and the communication interaction between individuals. However, when multiple robots cooperate, the data storage of most existing swarm robotics systems relies on the central node [6,7], which may cause a potential security threat of a single-point of failure as well as the incompletion and unreliability of the data.

When swarm robots are used in emergency scenarios, the system stability and data security of swarm robotics are key issues that need to be solved, and the stability of the swarm robotics system and the security of data are also key issues that need to be solved. First, due to the complex and changeable environment in the disaster scenarios, swarm robots should have autonomy and mobility to meet the system functions and performance requirements, resulting in complex network structure and unstable topology of heterogeneous swarm robots. Second, the changeable topology of the emergency scenarios causes the change and instability of the communication channel. In order to ensure the relative stability of the function and performance of the support system, swarm robots need to exchange a large amount of information and is highly dependent on the reliability of the information. Thus, ensuring the security of the communication data of swarm robots has become one of the key research topics for the rapid building of rescue support systems for swarm robots.

Blockchain, as a new distributed infrastructure and computing paradigm, was first used in the field of cryptocurrency to ensure the security and credibility of asset transfer [8]. It has the characteristics of data integrity and non-tampering, among others [9].The consensus algorithm plays a key role in the blockchain becoming a decentralized system, and its role is to enable a decentralized system with highly decentralized decision-making to efficiently and quickly agree on the validity of data. From the perspective of network structure, swarm robotics and blockchain have the same characteristics, and both have the characteristics of decentralization. Thus, blockchain technology can provide a new solution to the communication data security problems in heterogeneous swarm robotics systems. The blockchain is a decentralized database that can establish trust relationships in communication networks without central nodes [10]. For example, Abhi A.I. [11] proposed a secure data collection scheme based on blockchain technology. Robot data is collected from IoT devices using drone swarm and stored in the server’s blockchain to ensure the integrity of data collected by robots. The data in the method is transmitted in a publicly visible manner, and the confidentiality of the data is guaranteed. Kapitonov A. [12] proposed a multi-agent systems communication protocol based on blockchain, which ensures the communication security of UAVs in multi-agent system, but the protocol does not solve how to achieve consistency in the UAV data. Strobel V. [13] established a secure collaborative robot in swarm robotics by using smart contracts in blockchain technology to identify and exclude robot nodes with malicious behavior, but this method led to a decrease in the communication speed of the swarm robots, and the swarm robotics computing and storage capacity is limited.

According to the aforementioned literature, blockchain technology has solved some of the data security problems of robotics, such as ensuring the reliability of data or the identity authentication of robot nodes. However, in emergency rescue scenarios, due to the mobility of swarm robotics and the unreliability of communication links, if the blockchain technology is directly applied to the construction of heterogeneous swarm robotics systems, the following problems still need to be solved. First, swarm robotics has limited resources. Due to the changeable network topology of the heterogeneous swarm robotics system, the robotics system needs to generate a large amount of control data to maintain the stability of system functions and performance. However, due to limited resources, swarm robotics is not suitable for storing robot communication data and control data of the system. Second, there is Byzantine generals problem in the heterogeneous swarm robotics system. The mobility of the swarm robots that builds the rescue support system leads to the change of the topology of the scene, which causes the change and instability of the communication channel, resulting in unreliable communication data and control data between robots in the system.

In response to these challenges, we proposed a decentralized heterogeneous swarm robotics data security communication scheme for robots in emergency scenarios. The scheme ensures the stability of the heterogeneous swarm robotics system and the security of data in emergency scenarios. The contributions of this paper are summarized as follows.

- We proposed a decentralized network topology model, which is mainly composed of base robot, communication robots, and business robots. The decentralized control model is adopted to ensure the stability of rescue support system.
- We designed a data storage model based on the master–slave chains. This model divides the network into different slave chains according to different types of business robots. The slave chains mainly reach the consensus of local data; the data abstracts stored in all slave chains are uploaded to the master chain composed of base robot and communication robots to reach a system global consensus. This model adopts the Delegated Proof of Stake (DPoS) consensus mechanism to complete the consensus task of robot data so that the system can guarantee the consistency of data without a central node.
- We provided an implementation framework for the data communication scheme based on the main side chain and verified the scheme from the aspects of delay, throughput, and fault tolerance. The simulation results show that the performance of the data communication scheme based on the master–slave chains method is obviously better than that of the data communication scheme based on the single-blockchain method, and the scheme also has higher fault tolerance.

The remainder of this paper is structured as follows. Section 2 introduces the research background of the application of heterogeneous swarm robots. Section 3 gives an overview of the related work of blockchain technology. Section 4 describes the system model of the data communication using the master–slave blockchain. Simulations are given in Section 5 to evaluate the performance of the data communication using the master–slave blockchain. Finally, Section 6 draws the conclusions and presents future work.

## 2. Background

### 2.1. Heterogeneous Swarm Robotics Network Topology

**Figure 1:**
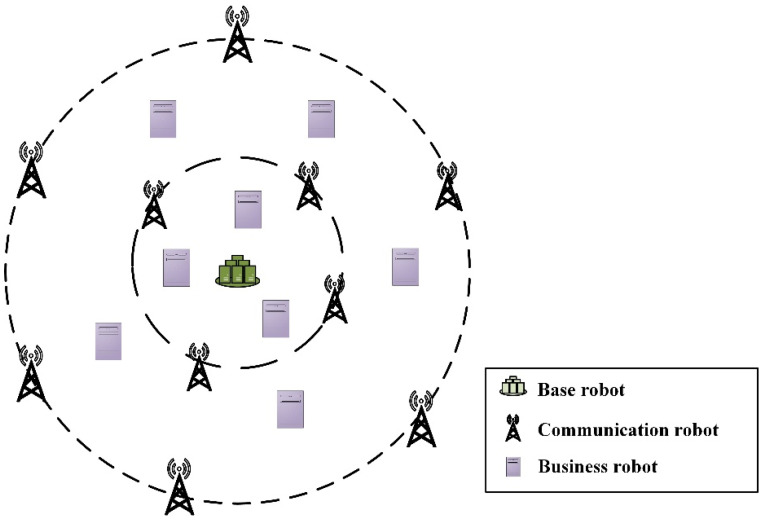
Network topology diagram of heterogeneous swarm robotics system.

After the disaster, the original environment of the disaster area is changed or the basic communication facilities are destroyed, the whole post-disaster environment is in an unknown state, and the system relying on the communication infrastructure communication facilities is paralyzed and cannot be used. However, the traditional communication strategies that rely on humans to complete the deployment of communication networks are limited by the geographical environment, and it is difficult for rescuers to enter the disaster-stricken area [14]. Thus, mobile robots are used to enter an unknown environment, conduct an initial exploration of the environment, collect the location of survivors through search, and provide accurate information for subsequent rescuers and improve rescue efficiency. However, the common rescue robots at present are mostly single robots [15], and their environmental adaptability is poor. In complex environments, multiple robots cannot cooperate effectively, and computing resources cannot be shared, making it difficult to complete rescue tasks. Thus, mobile robotics is used to quickly build an emergency rescue security system, including but not limited to communication service capabilities and computing service capabilities. Due to the uncertainty and complexity of the environment, in order to ensure the robustness of the rescue support system, we adopt a heterogeneous method to quickly build an emergency rescue support system. The network topology of the heterogeneous swarm robotics is shown in Figure 1.

### 2.2. Problem Description

The rescue support system is built by heterogeneous swarm robots, which adopts a centralized control method, that is, multiple business robots collect data, and the collected data is transmitted to the base station robot for storage through direct transmission or transfer by communication robots. Heterogeneous swarm robots can effectively deal with various complex environmental constraints to a certain extent, but emergency scenarios assisted by heterogeneous swarm robots have the following challenges:

- Due to the complex and changeable environment in the variable area, in order to ensure the function and stability of the system, the mobility and autonomy of the heterogeneous swarm robots lead to the complex network structure of the system and the unstable communication channel. The heterogeneous swarm robots use wireless communication for data interaction, and the robots are prone to data missing or errors during the communication process, which affects the normal operation of the system.
- The heterogeneous swarm robots system is based on a centralized data storage architecture, that is, the data collected by the business robot is uploaded to the base robot directly or through the communication robot for data storage. However, the swarm robots in this system have autonomy and swarm behavior control. After the whole system is completed, the control right in the system belongs to the robot itself, and the emergency rescue environment is uncontrollable, and its complex and changeable geographical environment can easily affect the normal operation of swarm robots to varying degrees. Thus, once a robot node fails, the entire heterogeneous swarm robotics system will be paralyzed.
- To this end, we describe the problem in the heterogeneous swarm robot system as a Byzantine generals problem [16]. There are N robots in the heterogeneous swarm robotics, assuming the following conditions:
  ○ Treat the normally operating robots in the system as loyal generals and complete their own tasks in accordance with the rules defined by the system;
  ○ The faulty robot in the system is regarded as a traitorous general, that is, the robot node fails or is attacked in the process of completing the task;
  ○ The N robots in the system communicate with each other.

At the same time, the core problem of this research is how to find a “solution” in the complex and changeable emergency environment so that the constructed rescue support platform can rely on this “solution” to protect the integrity and confidentiality of the swarm robotics data and availability, improve the robustness of the communication network of the whole system, and realize the safe operation of the communication network in the system. The emergence of blockchain technology provides a new solution to this problem.

## 3. Related Work

The robots in the heterogeneous swarm robotics system have autonomy and mobility. Multiple robot nodes perceive their surrounding environment through the sensors they carry and perform data transmission, calculation, and processing [17]. The distributed network structure can effectively improve the stability of the heterogeneous swarm robotics system. However, how to ensure data security between multiple robots has always been a research hotspot [18].

Higgins F. [19] summarized the security problems of swarm robotics and identified the following three challenges. First, there are robot nodes that are attacked or fail: messages sent from these nodes may contain errors or deceptive information. The second is the instability of the communication channel: the information in the swarm robots is transmitted through the point-to-point network, which is prone to error in the process of transmission. Third, robot nodes lose usability; the information stored in the robot is deleted, resulting in the destruction of the robot.

As a distributed data storage and security management method, blockchain technology provides a new solution for data security problems in distributed systems. For example, Roy S. [20] proposed a cloud framework–based IoT security and computing management method for multi-robot systems in rescue operations and uses blockchain technology to ensure the security of robots in clusters. The blockchain technology is used in the Rover, which has low scalability. Zhang X. [21], based on the traffic signal control mechanism of the alliance blockchain, used the group signature scheme as the trusted mechanism to provide a secure and reliable communication environment for VANET, but this method mainly aims at the traffic safety in the Internet of Vehicles, and is not directly suitable for the emergency scenarios with unknown environments. Xie L. [22] proposed a blockchain-based VANETs trust model which utilizes the features of blockchain decentralization and non-tampering to ensure the security and privacy issues in the vehicle IoT environment, but the computational overhead of this method is too high. Lu Y.X. [23] proposed a hybrid blockchain architecture to solve the data security problem in the Internet of Vehicles. The algorithm needs to learn model parameters locally through federated learning, which requires high computing power of participating nodes. Jiang Y. [24] proposed a decentralized data sharing solution for an IIOT network based on blockchain and edge computing. With the help of edge computing, the distributed consistency of blockchain network and the distributed storage of shared data are realized at the edge of the IIoT network, which is not suitable for the scenario of node mobility. These studies address the security of data in distributed system, but how to ensure the consistency of data among multiple nodes in a distributed system is also a problem that needs to be solved.

As the core technology of the blockchain network, the consensus algorithm can ensure data consistency among multiple nodes in the distributed system [25]. Queralta J.P. [26] proposed a block chain–based collaborative management method for heterogeneous swarm robots to ensure that heterogeneous swarm robots can share information through collaboration without sharing identity and computing resources. In this method, a method of combining proof of work (PoW) consensus mechanism with slicing technology is proposed to improve the scalability of the system. However, there is a waste of computing resources in this method. Singh P.K. [27] proposed a new distributed collective decision-making algorithm for the safety of distributed collective decision-making of robots in swarm robotics to ensure the safety of the collective decision-making of swarm robots. However, the above studies all use the PoW consensus mechanism, which has the problem of wasting computing resources. Pacheco A. [28] designed an AdHoc communication network for swarm robots controlled by blockchain, which ensures that the consensus mechanism of the proof-of-authority mechanism is adopted to reduce the computational cost of robots and realizes the effective operation of the swarm robotics system based on blockchain. However, the proof-of-authority mechanism consensus protocol lacks sufficient analysis [29]. Alsamhi S.H. [30] proposed a new consensus algorithm to ensure that in a decentralized UAV network, multiple UAVs can quickly reach agreement for cooperative work. Secondly, the application of fragmentation technology can improve the scalability of UAV networks. Jiang L.S. [31] proposed a consensus mechanism for the proof of entrusted rights and interests. The algorithm introduces the consensus criterion of the “election mechanism”. The nodes with rights and interests choose n trust nodes by voting as the consensus process in the system, and each trust node becomes an accounting node in turn within a fixed period.

Through the analysis of the aforementioned literature, we found that the existing solutions based on the application of blockchain technology to solve data security problems in a distributed system can only solve some problems and are not suitable for the emergency rescue scenarios studied in this paper. To this end, we propose a data communication model of rescue support system based on the master–slave chains to solve the data security problem of heterogeneous swarm robots in emergency scenarios.

## 4. System Model

### 4.1. Decentralized Heterogeneous Swarm Robotics Data Security Communication Model

The heterogeneous swarm robotics system is composed of three types of robots: base robot, communication robots, and business robots. Among them, the base robot has high computing resources and communication resources and is responsible for the identity registration of the legal robots entering the swarm robotics network, generating public and private key pairs, and issuing identity certificates. The communication robots are distributed in the middle of the detection edge of the emergency scene, which can be connected to the base station upward through the wireless communication link and connected downward to the business robot or communication robot within its coverage. The business robot performs related rescue tasks and can communicate with other robots in the system.

**Figure 2:**
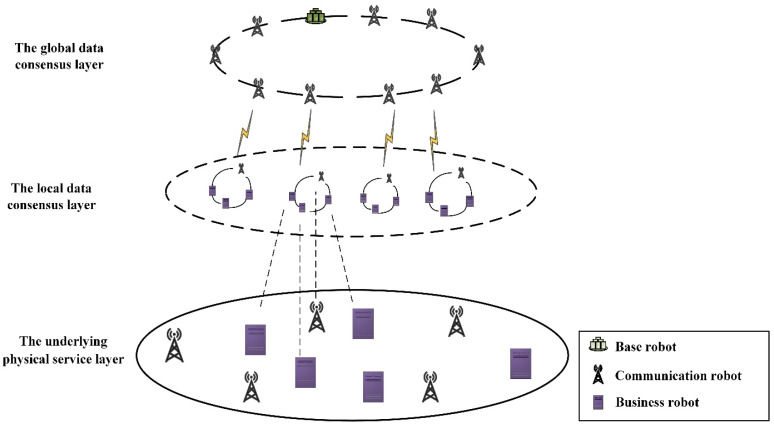
The decentralized heterogeneous swarm robotics communication model.

Due to the complex and changeable network topology of the heterogeneous swarm robotics system, the communication channel is unstable, and data security problems are caused. Thus, we proposed a decentralized heterogeneous swarm robotics communication model, as shown in Figure 2. The model is divided into three layers, from bottom to top: the underlying physical service layer, the local data consensus layer, and the global data consensus layer.

The underlying physical service layer: refers to the underlying robot node. It mainly includes base robot, communication robots, and business robots. In the underlying physical service layer, all kinds of robot nodes can act as data senders and data receivers. Robot nodes reach global data consensus or local data consensus.

The local data consensus layer: refers to the blockchain composed of the same type of business robots combined with industrial communication robots. There are multiple slave chains in the rescue support system. Different slave chains can interact through the main chain. The main function is to maintain the transaction information of all robots on the chain where it is located and to achieve data consistency among all robots on the chain.

The global data consensus layer: refers to the blockchain composed of base robot and communication robots with high computing, communication, and storage resources. It is the only one in the rescue support system and mainly stores the data on the slave chains. The hash digest completes the global consensus of the rescue support system ensures the consistency of all robot data in the system and facilitates subsequent data verification.

In the decentralized heterogeneous swarm robotics communication model proposed in this paper, a dynamic networking method is adopted in this model, and the robots are connected to the network one by one during the operation of the system. There is a distributed heterogeneous swarm robotics network, on the basis of which the robot nodes in the group process the data in the system using hierarchical consensus to achieve data consistency. Secondly, the distributed communication network and the method of hierarchical consensus enhance the scalability of the swarm robot system to a certain extent.

### 4.2. Decentralized Heterogeneous Swarm Robotics Network Model

The decentralized heterogeneous swarm robotics network model mainly completes robot node identity authentication, robot node access, and blockchain networking control. The descriptions of the symbols involved in this subsection are shown in Table 1.

**Figure 3:**
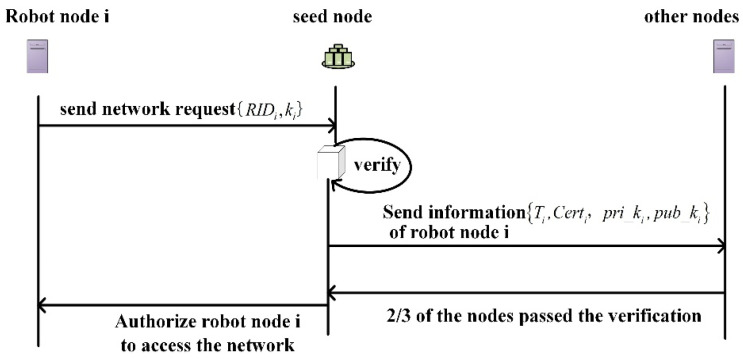
Networking process of rescue support system based on blockchain.

In this paper, dynamic networking is used in the system initialization stage for networking, *k*. Before the system runs, the base robot in the system is used as a seed node, and the networking rules are determined. In the system initialization stage, each robot node is connected to the network one by one and autonomously forms a team to build the main chain. When each subsequent robot node joins the network, it needs a method of authorization based on the seed node and the identity authentication of most robot nodes in the network. The networking process is shown in Figure 3.

The specific steps of system initialization are as follows:

Step1: Parameter setting. Before the system runs, the base robot is artificially designated as the seed node in the system, which is responsible for storing and managing the initial information of the network access node and the issuance of the identity certificate;

Step 2: The robot node i sends a network access request $\left\{RID_{i},k_{i}\right\}$ to the seed node;

Step3: After the seed node receives i the network access request from the robot node, it authenticates i the identity of the node, generates a joining authorization certificate after successful authentication $Cert_{i}$, selects an elliptic curve Ep(a,b), and takes a point on the elliptic curve as the base point G(x,y). The robot node i selects the sent $k_{i}$ private key $pri\_k_{i}$ as the robot node i and generates the node’s public key $pub\_k_{i}=pri\_k_{i}\times G\left(x,y\right)$. Then, the seed node broadcasts the registration timestamp of $T_{i}$, the robot node i, and other nodes in $pub\_key_{i}$ the master chain;

Step4: If two-thirds of the robot nodes $\left\{T_{i},Cert_{i},pri\_k_{i},pub\_k_{i}\right\}$ pass the authentication on the i transaction broadcast by the seed node, the robot node is authorized to enter the network, and the node is successfully connected to the network.

### 4.3. Data Storage Model of Heterogeneous Swarm Robotics Using the Master–Slave Blockchain

The data storage model of heterogeneous swarm robotics using the master–slave blockchain consists of two parts: the design of the master–slave blockchain blocks and the consensus mechanism based on the data storage of the master–slave blockchain. It mainly completes the decentralized storage of robot information in the heterogeneous swarm robotics system and completes the local consensus or global consensus in the system to ensure the data consistency of multiple robot nodes in the system.

#### 4.3.1. Design of the Master–Slave Blockchain’s Block

##### The Data Structure of the Slave Chains Block

The slave chains are composed of business robots and communication robots, which mainly store the complete data information of the business robots in the process of performing tasks and reach a consensus of all nodes in the slave chains. The block header structure of the slave chains is shown in Table 2.

**Figure 4:**
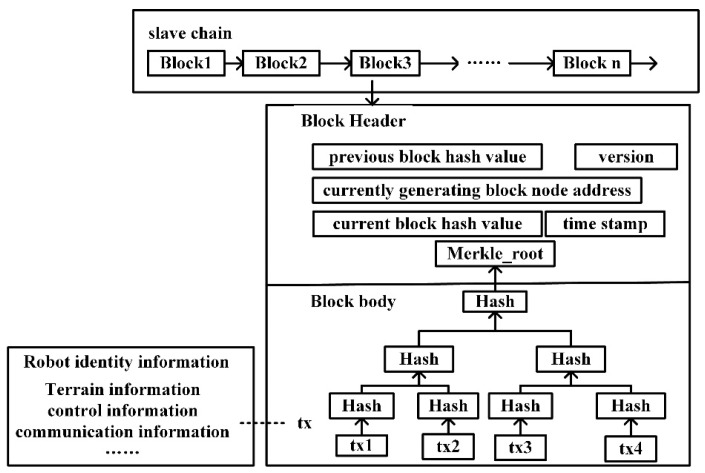
Data structure of the slave chains block.

The block of the slave chains stores the transaction data information generated by the business robot on the chain, including robot identity information, terrain information, communication information, control information, etc. Different types of business robots form different slave chains. For the data in the slave chains block body, the hash value is taken up in turn according to the method of pairwise hashing until the hash value is taken for the Merkle root again and stored in the block header. When any data in the slave chains block changes, the hash value of the Merkle root in the block header also changes, thus ensuring the immutability of the slave chains block data. The data structure of the slave chains block is shown in Figure 4.

##### The Data Structure of the Master Chain Block

**Figure 5:**
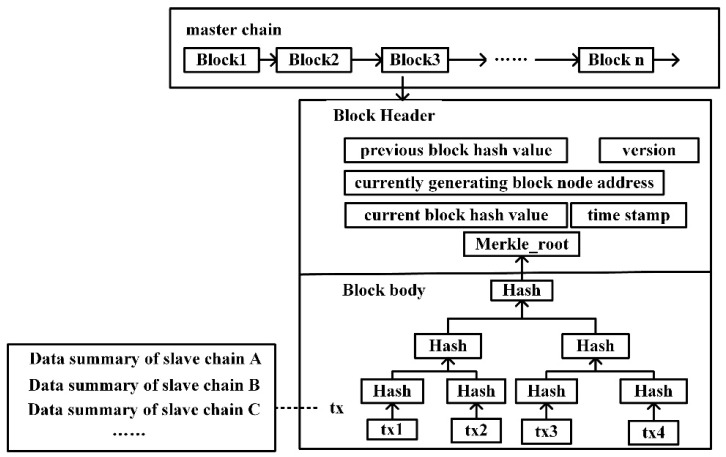
Data structure of the master chain block.

The master chain consists of a base robot and a communication robot. It mainly stores the hash value of the stored data on all slave chains, and reaches the consensus of the entire network of the rescue support system, so as to facilitate the search and verification of robot data in the subsequent system. For the hash value of the slave chains data in the master chain block, the hash function is still used to take the hash value up in turn in a pairwise hashing manner, until the Merkle root is taken once again, the current value of the Merkle root is salved in the block header of the block generated by the master chain block. The data structure of the master chain block is shown in Figure 5.

When the business robots in each slave chains initiate a data storage transaction to the slave chains maintained by itself, the communication robots in the slave chains pack the data into a block, and, at the same time, the communication robot also uploads a digest of the data-packaged data to the master chain for storage and broadcast on the whole network, which is helpful for the query and verification of all of the network nodes.

##### 4.3.2. Consensus Mechanism Based on Master–Slave Blockchain Data Storage

First of all, considering the limited battery capacity of swarm robots and the inability to charge during task execution, how to reduce resource consumption is one of the difficult problems to be solved in reaching an information consensus. Second, shortening the communication time between nodes is also necessary to ensure that swarm robots cooperate efficiently to perform tasks and avoid collisions. As the most balanced mechanism among the mainstream consensus mechanisms, the DPoS consensus algorithm not only has the characteristics of low confirmation delay, low resource consumption, and high scalability but also has high throughout [32]. Therefore, the DPoS consensus mechanism is used as the consensus mechanism for the data storage model of heterogeneous swarm robotics using the master–slave blockchain to improve the consensus efficiency of swarm robotics. The DPoS consensus algorithm mainly includes the following parts:

###### Node Type

The model of the proxy node in this paper includes three roles: common node, candidate node, and proxy node.

The common node refers to the business robot node that occupies the largest proportion in the system. Due to its limited resources, this type of robot cannot support the block generation and verification process. Therefore, it only has the right to vote. Candidate nodes refer to the base robot and communication robots in the system. These two types of robots have the right to vote and be voted because they can complete the generation and verification of blocks due to their sufficient resources. The proxy node is a set of accounting nodes jointly elected by ordinary nodes and candidate nodes and has the right to generate blocks and verify blocks

###### Proxy Node Election Mechanism

In the master and slave chain consensus mechanism based on DPoS designed in this paper, all robots in the rescue support system serve as blockchain users, forming the master–slave chains in the system. In the process of proxy node election, blockchain users vote for the candidate nodes they support by using their own equity as the number of votes, and each voting node can vote for other candidate nodes. When the voting is over, the system counts the votes of each full node and designates the top N candidate nodes with the highest votes as the set of proxy nodes for the current cycle.

###### Proxy Nodes Produce Blocks

In the DPoS consensus mechanism, each proxy node in the set of proxy nodes selected in the proxy node election mechanism completes the rights to produce blocks and verify blocks in the master and slave chains to ensure data consistency. Each proxy node takes turns to package the transaction information in the master chain/slave chains transaction pool into a new block within a specified time, and then broadcast it to other proxy nodes. If there is a proxy node that fails to produce blocks on time, the proxy node is skipped, and the next proxy node continues to produce blocks, which can effectively avoid the system delay caused by the failure of a proxy node to account in time due to its own failure.

###### Block Verification

In the DPoS consensus mechanism, whenever a proxy node generates a new block within the specified time, it broadcasts the block to other N-1 proxy nodes in the set of proxy nodes. The time stamp and transaction information in the block are verified, and after the verification is passed, successful verification information is fed back to the proxy node of the block. When the proxy node producing the block receives feedback from 2N/3 other proxy nodes, it adds the new block to the blockchain it maintains and broadcasts it to other robot nodes for storage.

**Figure 6:**
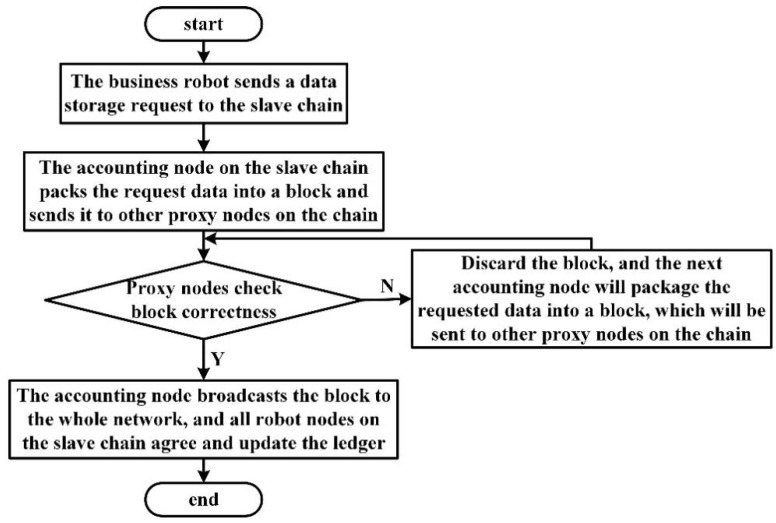
Robot data consensus process of slave chains.

In the data storage model of heterogeneous swarm robots using the master–slave blockchain, the consensus process of the whole system is divided into local data consensus and global data consensus. In local data consensus, business robots and communication robots on the same slave chains interact with each other and store transaction data on the slave chains to reach a local consensus. In the global consensus, the communication robots on multiple slave chains upload the digests stored on the chains to the main chain, and the robot nodes of the main chain share them to reach a global consensus. In the data storage process based on the main and slave chains mentioned in this paper, the consensus content of the slave chains and the main chain is similar. For the convenience of analysis, this paper only gives the consensus process of the slave chain layer. The data consensus process of swarm robotics on the slave chains is shown in Figure 6.

The business robot in the slave chains collects various data information in emergency scenarios. Once it needs to interact with other robots, it initiates a request to the slave chains. After the data request is made by the accounting node of the slave chains, the request data is packaged and broadcast to other proxy nodes on the slave chains to verify the transaction data in the received block, and the verified block is added to the slave chains as well as broadcast to other robot nodes on the slave chains for storage. The DPoS consensus algorithm based on the master–slave chain data storage model is shown in Algorithm 1.
**Algorithm 1** DPoS consensus algorithm based on master–slave blockchain data storage**Input:** Common node set $A_{1}=\{x_{1},x_{2},\cdots ,x_{n}\}$, candidate node set $A_{2}=\{x_{1},x_{2},\cdots ,x_{l}\}$**Output:** Datastore success or datastore failure1. Common nodes and candidate nodes select the proxy node set $R_{m}=\{x_{1},x_{2},\cdots ,x_{m}\}$
by voting2. $M_{i}\in R_{m}$
3.
$M_{i}$
package block information
4. $M_{i}$ broadcast message(block, blockMessage)
5. Other proxy nodes verify blocks
6. If the block verification is passed and the cumulative number of verification passes is 2N/3

7.   Message
$\left(block_{true}\right)$ broadcast by node $M_{i}$ and add the block to the slave blockchain
8. Else
9.   Data storage failure message broadcast by the node $M_{i}$10. end if

## 5. Evaluation

In order to verify the feasibility of the model designed in this paper, a prototype of a master–slave chain heterogeneous swarm robotics system is implemented based on the golang language, and the master–slave chains system is deployed into the Docker container to simulate more nodes for the data storage process on the chain. The system is tested in the test environment, and the test environment configuration setting was set up as stated in Table 3.

### 5.1. Performance Analysis of the System

In order to test the performance of the prototype of the heterogeneous swarm robotics system based on the master–slave chains constructed in this paper, throughout and consensus delay are used to measure the performance in the process of data uploading and storage. Throughout refers to the number of transactions completed in unit time, and consensus delay refers to the time consumed from transaction submission to transaction completion. In the heterogeneous swarm robotics system based on the master–slave chains proposed in this paper, both the master chain and the slave chains adopt the DPoS consensus mechanism. In the case of the same number of robotics systems, the heterogeneous swarm robotics system based on the single chain and the heterogeneous swarm robotics system based on the master–slave chains are compared, respectively. The experimental configuration is shown in Table 4.

**Figure 7:**
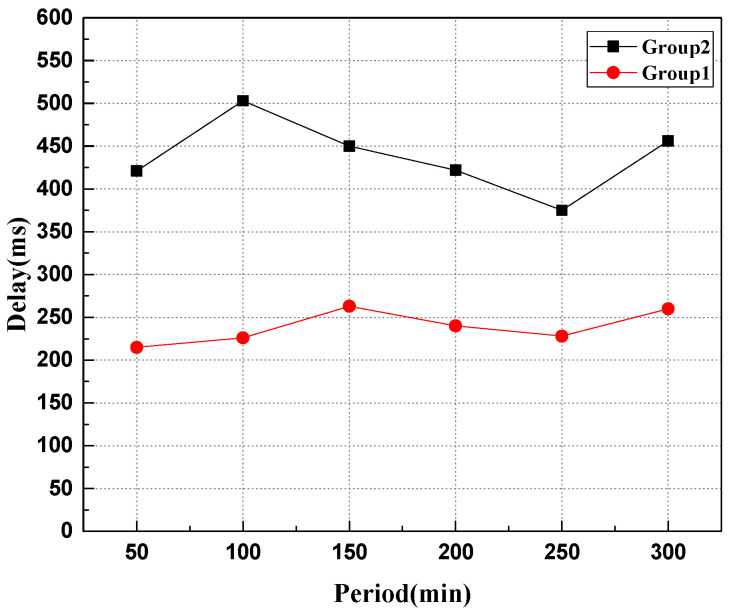
Comparison of system delay during data uplink.

**Figure 8:**
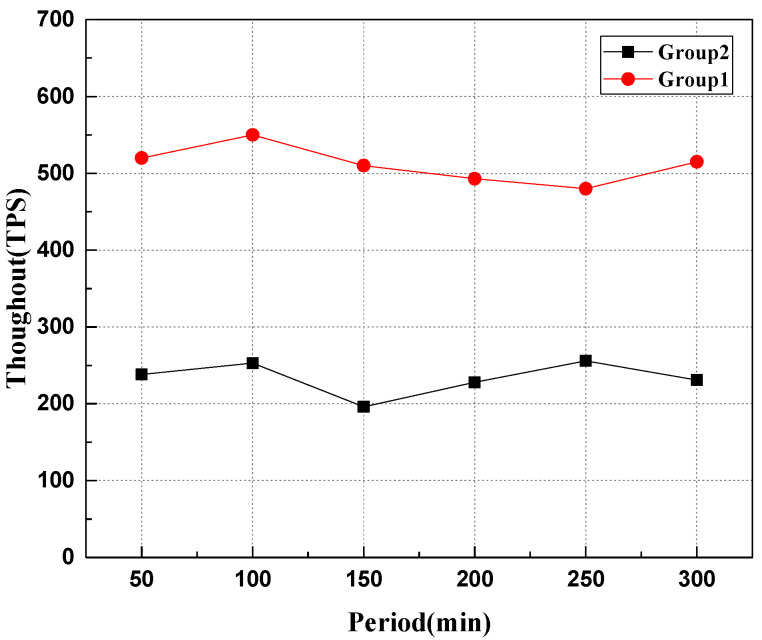
Comparison of system throughout during data uplink.

This experiment simulates that the robot node continuously sends transactions to the system, in which experiment group 1 sends data storage requests to the entire network nodes, and experiment group 2 continuously sends data storage transactions to the two slave chains to test different configurations. The performance of the system and the experimental results are shown in Figure 7 and Figure 8.

As can be seen from Figure 7, when the data of the robot nodes are the same, the delay of the heterogeneous swarm robotics system based on the master–slave chains is significantly lower than that of the heterogeneous swarm robotics system based on the single chain in the process of data uploading. The reason is that in the model using the master–slave blockchain, the consensus of the block only reaches a local consensus on its own chain, while in the single-chain model, when the data is stored on the chain, all nodes on the chain need to synchronize the data to reach a global consensus. The more nodes that need consensus, the more time it takes.

As can be seen from Figure 8, when facing different transaction data requests, the transaction throughout of the heterogeneous swarm robotics system based on the master–slave chains is roughly maintained at about 500 transaction/s, while it is at about 230 transaction/s based on the single chain. The multi-slave chains in the heterogeneous swarm robotics system based on the master–slave chains can run in parallel, so the overall throughout of the system is improved. According to the experimental data, the decentralized swarm robotics system based on the master–slave chains is more suitable for emergency scenarios.

**Figure 9:**
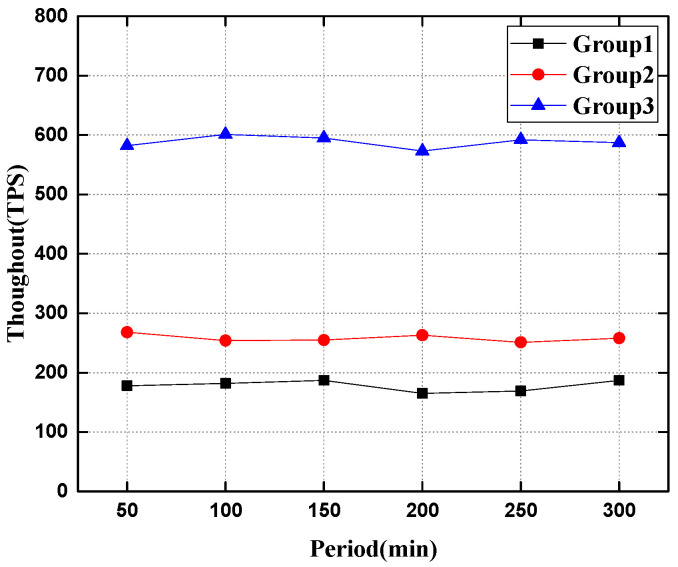
Comparison of system throughput performance when the number of slave chains is different.

In order to further simulate and verify the influence of the number of side chains on the system performance of the heterogeneous swarm robotics data communication system of the main side chain proposed in this paper (that is to say, when the number of side chains is different in the test prototype system), the throughput is used to measure the performance index. To measure the performance of the heterogeneous swarm robotics communication system based on the main side chain, the experimental configuration is shown in Table 5, and the experimental results are shown in Figure 9.

As can be seen from Figure 9, in the heterogeneous swarm robotics system using the master–slave blockchain, the performance of the system has been greatly improved in terms of transaction throughout with the increased number of slave chains in the system. Because in the model using the master–slave blockchain, when a robot node initiates a transaction request, it first reaches a local consensus on the side chain and stores the data, and then the nodes on the slave chains send an upload request to the master chain to reach a global consensus on the data. Therefore, when the number of slave chains in the heterogeneous swarm robotics system is greater, the performance of the heterogeneous swarm robotics prototype system using the master–slave blockchain shows an upward trend. It can be seen from the experimental simulation results that the decentralized swarm robotics system using the master–slave blockchain can process data quickly and achieve data consistency among multiple robots in the case of a large amount of data.

### 5.2. Fault Tolerance Analysis

**Figure 10:**
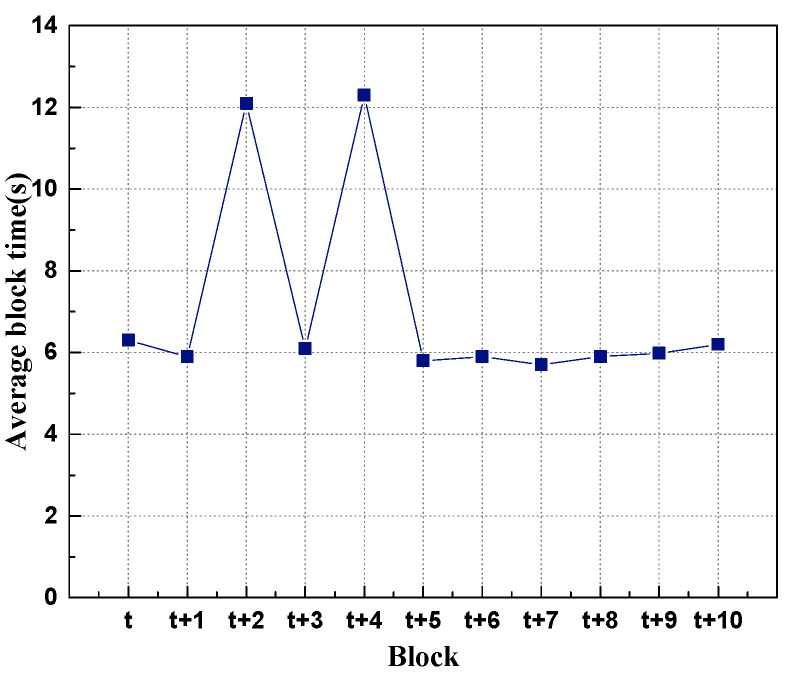
Fault tolerance experiment.

During the operation of the prototype system, the proxy node is in an abnormal state to simulate the failure of the robot node so as to simulate the fault tolerance of the prototype system when the robot node in the prototype system is abnormal and obtain experimental data for analysis and experiment. The results are shown in Figure 10.

From the experimental results, when a proxy node in the system fails, the block generation time of the next block increases to about twice the original time until the faulty proxy node generates a block. When the node returns to normal, the next block returns to the normal block time. Although there is a fault in the system, it affects the block generation time of the node, and it can still ensure the normal operation of the system. Therefore, the decentralized heterogeneous swarm robots based on the master–slave chains has strong fault tolerance.

## 6. Conclusions

In this paper, a decentralized heterogeneous swarm robotics data communication scheme was presented for emergency scenarios. First, the scheme established a decentralized heterogeneous swarm robotics network topology which solves the instability of the heterogeneous system. Second, we proposed a data storage model using the master–slave blockchain, in which the DPoS consensus mechanism is used to ensure the consistency of robot data when the central node is not fixed. In addition, we provided an implementation framework of the data security communication scheme using the master–slave blockchain, and the effectiveness of the scheme was verified by simulation from the aspects of delay, throughput, and fault tolerance. In the future, the scheme proposed in this paper will be further improved and perfected to enhance its applicability to unknown environments so as to further improve the scalability and stability of the scheme.

## Figures and Tables

**Table 1:** Symbol description.

Symbol	Explain
$\left\{RID_{i},k_{i}\right\}$	internet request
$k_{i}$	nonce
$Cert_{i}$	certificate of authorization
$pri\_k_{i}$	robot private key
$pub\_k_{i}$	robot public key

**Table 2:** The block header data structure of the slave chains block.

Constitute	Explain
Version	version number
Height	block height
Timestamp	timestamp
Previous Block Hash	slave chains previous block hash
Merkle Root	The hash of the Merkle root in the slave chains blockchain

**Table 3:** The test environment configuration parameters.

Operating System	Linux	Ubuntu18.04.6 LTS
**Hardware Configuration**	kernel	Kernel 4.15.0-161-generic
	CPU	Intel(R) Xeon(R) CPU E5-2620 v4 @ 2.10 GHz
	hard disk	3.7 T
	Memory	16 G
**Software configuration**	Docker	20.10.17
	GCC	7.5.0
	Ethereum	1.7.4
	Golang	1.17.11

**Table 4:** Experimental configuration table.

Experimental Group Configuration	Group 1	Group 2
consensus algorithm	DPoS/DPoS	DPoS
system model	Master–slave chains	single chain
number of main chain nodes	10	-
number of slave chains nodes	2/10	-
total number of nodes	20	20

**Table 5:** Experimental configuration table.

Experimental Group Configuration	Group 1	Group 2	Group 3
number of main chain nodes	20	20	20
number of slave chains	1	2	5
number of slave chains nodes	20	10	4
total number of nodes	40	40	40

## Data Availability

Not applicable.
